# Maternal antenatal folate levels and offspring risk of IgE-mediated food sensitisation

**DOI:** 10.1186/2045-7022-5-S3-P90

**Published:** 2015-03-30

**Authors:** John Molloy, Richard Saffery, Jennifer Koplin, Anne-Louise Ponsonby, Mimi LK Tang, Fiona Collier, Katrina Allen, Peter Vuillermin, BIS Investigator Group

**Affiliations:** 1 Child Health Research Unit, Barwon Health, Geelong Hospital, Victoria, Australia; 2 Murdoch Children's Research Institute, Parkville, Australia; 3 School of Medicine, Deakin University, Waurn Ponds, Australia; 4 University of Melbourne, Parkville, Victoria, Australia; 5 Department of Allergy and Immunology, Royal Children's Hospital, Parkville, Victoria, Australia

## Background

The prevalence of IgE-mediated food allergy has increased in the developed world, particularly among children less than 5 years of age. Widespread folate supplementation during pregnancy commenced in the 1980's in an attempt to decrease the rate of neural tube defects. This introduction corresponded with the increase in inflammatory disease in children, including allergic conditions. Elevated maternal folate has been associated with allergic disease development in mice, yet there is conflicting evidence in humans as to whether elevated levels, associated with folic acid supplementation in pregnancy, may be associated with allergic airway disease in children.

## Objective

To determine if maternal red-cell folate levels in the third trimester of pregnancy are associated with food sensitization in infancy in a population-derived cohort of infants.

## Methods

The Barwon region is located 100 km southwest of Melbourne (Australia) with an urban centre and rural hinterland. The Barwon Infant Study (BIS) is a population-derived birth cohort study (n = 1069). As part of the 12 month review infants undergo skin prick testing to five foods: cow's milk, raw egg, peanut, cashew and sesame. Those with any detectable wheal size are offered a formal in-hospital oral food challenge using validated protocols to determine their clinical allergy status. Red cell folate levels were obtained from pregnant women in the study at 28 weeks gestation.

## Results

692/1069(64.7%) women had red cell folate measured during the third trimester of pregnancy. The mean red cell folate level in the cohort was 1727.18 nmols/litre (nmols/L). Only 5 women had a folate level below 900 nmols/L. The mean red cell folate level in mothers of non-sensitized infants was 1716.65 nmols/L (95% CI, 1636.82-1796.49), whereas the mean level in mothers of sensitized infants was 1839.05 nmols/L (95% CI, 1724.53-1953.572). Although elevated in the sensitized group, the difference of 122.39 nmols/L (95% CI, -385.0152-140.2213) was not statistically significant (p=0.3605).

## Conclusions

There was no association between maternal RBC folate levels during the third trimester of pregnancy and the offspring risk of food sensitization. However, these data are limited by the small number of mothers with lower levels of folate in the cohort, possibly associated with the widespread use of folic acid supplements in pregnant women.

